# HSPA6 is Correlated With the Malignant Progression and Immune Microenvironment of Gliomas

**DOI:** 10.3389/fcell.2022.833938

**Published:** 2022-02-23

**Authors:** Xiang Zhou, Qiankun Ji, Qin Li, Peng Wang, Guowen Hu, Feng Xiao, Minhua Ye, Li Lin, Min Luo, Yun Guo, Weijun Wu, Kai Huang, Hua Guo

**Affiliations:** ^1^ Departments of Neurosurgery, The Second Affiliated Hospital of Nanchang University, Nanchang, China; ^2^ Departments of Neurosurgery, The Fifth Affiliated Hospital of Nanchang University, Fuzhou, China; ^3^ Departments of General Practice, The Fifth Affiliated Hospital of Nanchang University, Fuzhou, China; ^4^ Institute of Neuroscience, Nanchang University, Nanchang, China; ^5^ Jiangxi Key Laboratory of Neurological Tumors and Cerebrovascular Diseases, Nanchang, China

**Keywords:** gliomas, heat shock protein family A member 6 (HSPA6), malignancy, experimental validation, tumor immune microenvironment

## Abstract

Gliomas are primary intracranial space lesions with a high mortality rate. Current treatments for glioma are very limited. Recently, immunotargeted therapy of the glioma microenvironment has been developed. Members of the 70 kDa heat shock protein (HSP70) family are involved in the development of many tumors and immunity. HSPA6 protein belongs to the HSP70 family; However, the biological function of this protein in gliomas has yet to be evaluated. In the present study, a range of analyses, involving protein networks, survival, clinical correlation, and function, revealed that the expression of HSPA6 was negatively correlated with clinical prognosis and closely associated with immunity, invasion, and angiogenesis. Quantitative protein analysis confirmed that HSPA6 was expressed at high levels in patients with glioblastoma. Vitro experiments further verified that HSPA6 enhanced the malignant progression of glioma cells by promoting proliferation, invasion and anti-apoptosis. We also found that HSPA6 was closely correlated with genomic variations and tumor microenvironment. Collectively, we demonstrated that HSPA6 may represent a new therapeutic target to improve the prognosis of patients with gliomas.

## Introduction

Gliomas are derived from a group of neuroepithelial tumors that are collectively known as brain gliomas and account for the vast majority of primary brain tumors, the most common form of primary intracranial tumors (Ostrom et al., 2014). The grading system developed by the World Health Organization (WHO) classifies gliomas from grade I (the least malignant and associated with the best prognosis) to grade IV (the most malignant and associated with the worst prognosis); of these, glioblastoma has the highest degree of malignancy and mortality (Weller et al., 2015; Jiang et al., 2016; Zhang et al., 2019). Currently, the most dominant treatments include surgery, temozolomide-targeted chemotherapy, and radiotherapy; however, the clinical prognosis of undergoing treatment for patients with these modalities is not satisfactory (Stupp et al., 2017). The investigation of gene/protein regulatory networks and immunopathological mechanisms may identify novel molecular targeting and immunotherapeutic methods for glioma (Eckel-Passow et al., 2015). Therefore, the discovery of new and effective treatments for glioma still remains an unprecedented challenge.

When heat shock proteins (HSPs) were first discovered in the salivary gland cells of *drosophila* in 1962 (Ritossa, 1962), they represent a widely distributed and highly conserved family of proteins that are usually expressed under a variety of physiological and stressful conditions, including carcinogenesis. According to molecular weight, the superfamily of HSPs can be divided into HSP110, HSP90, HSP70, HSP60, HSP40, and a family of small HSPs (Carper et al., 1987; Lindquist and Craig, 1988). Particularly, the ability of the HSP70 family to protect cells against stress (Beere and Green, 2001; Daugaard et al., 2007; Yang et al., 2012) is so efficient that they represent the most prominently conserved family (Oehler et al., 2000). Among humans, a total of 15 members of the HSP70 family have been detected at different sites, thus suggesting a range of biological functions that are location-specific (Daugaard et al., 2007; Rosenzweig et al., 2019). HSP70s are highly expressed in many tumors and promote the progression of malignancy by inhibiting apoptosis, evading cellular senescence-mechanisms, disturbing immunity, and promoting angiogenesis (Albakova et al., 2020). HSP70 proteins are highly expressed in a wide range of malignant cancers and are negatively correlated with a poor clinical prognosis (Calderwood et al., 2006). Beaman et al. (Beaman et al., 2014) proved that the expression of HSP70 genes and proteins were positively correlated with glioma grade. However, the mechanisms that mediate the effects of HSP70 family members on glioma remain unknown.

While investigating potential prognostic markers for human glioma, Sun et al. (Sun et al., 2020a) constructed a protein-protein interaction (PPI) network and found that nine heat shock proteins (DNAJA4, DNAJC6, DNAJC12, HSPA6, HSP90B1, DNAJB1, DNAJB6, DNAJC10, and SERPINH1) were differentially expressed; of these, only HSPA6 belonged to the HSP70 family. Other studies indicated that HSPA6 may have an inhibitory effect on bladder cancer (Shin et al., 2017), lung cancer (Wang et al., 2020a), and triple-negative breast cancer (Hahm et al., 2021; Shen et al., 2021), while may be relevant to the early recurrence of human hepatocellular carcinoma (Yang et al., 2015). However, the functional effect of HSPA6 in the progression of glioma remains unclear. In another study, Wang et al. confirmed that the expression of RAB34 conferred a poor prognosis in high-grade glioma patients; furthermore, HSPA6 was positively correlated with the expression of RAB34 (Wang et al., 2015). Therefore, we hypothesized that HSPA6 may be a reliable prognostic biomarker for gliomas.

In this study, we reconfirmed the core role of HSPA6, a member of the HSP70 family, by constructing a PPI network. To further predict the prognostic significance of HSPA6 in gliomas, bioinformatics analysis was performed on three different independent cohorts, including The Cancer Genome Atlas (TCGA), the Chinese Glioma Genome Atlas (CGGA), and the GSE16011 dataset. Survival analysis showed that the subgroup of patients of high levels of HSPA6 expression had a shorter survival time. This result indicated that HSPA6 may represent a biomarker of poor prognosis for patients with glioma and was further confirmed by analysis of time-dependent receiver operating characteristic (ROC) curves and area under the curve (AUC) values. By analyzing the clinical data, we detected relationships between HSPA6 expression and isocitrate dehydrogenase (IDH) mutations, 1p19q co-deletion, grade, gender, and age. These clinically relevant indicators were significant in both univariate cox regression analysis and multivariate cox regression analysis. Gene Ontology-Biological Process (GO-BP) analysis, Kyoto Encyclopedia of Genes and Genomes (KEGG) enrichment analysis, and Gene Set Variation Analysis (GSVA), further suggested that HSPA6 was mainly correlated with immunity, invasion, and angiogenesis. Compared with para-carcinoma tissues, HSPA6 protein was highly expressed in clinical samples of glioma. Furthermore, functional *in vitro* experiments verified the effect of HSPA6 in glioma cells and its possible pathway mechanism. Finally, comprehensive analyses revealed significant differences in genomic variation, tumor-infiltrating immune cells (TIICs), different tumor microenvironments (TMEs), immune checkpoint (ICP) expression levels, when compared between patients with low and high expression levels of HSPA6.

## Methods

**FIGURE 1:**
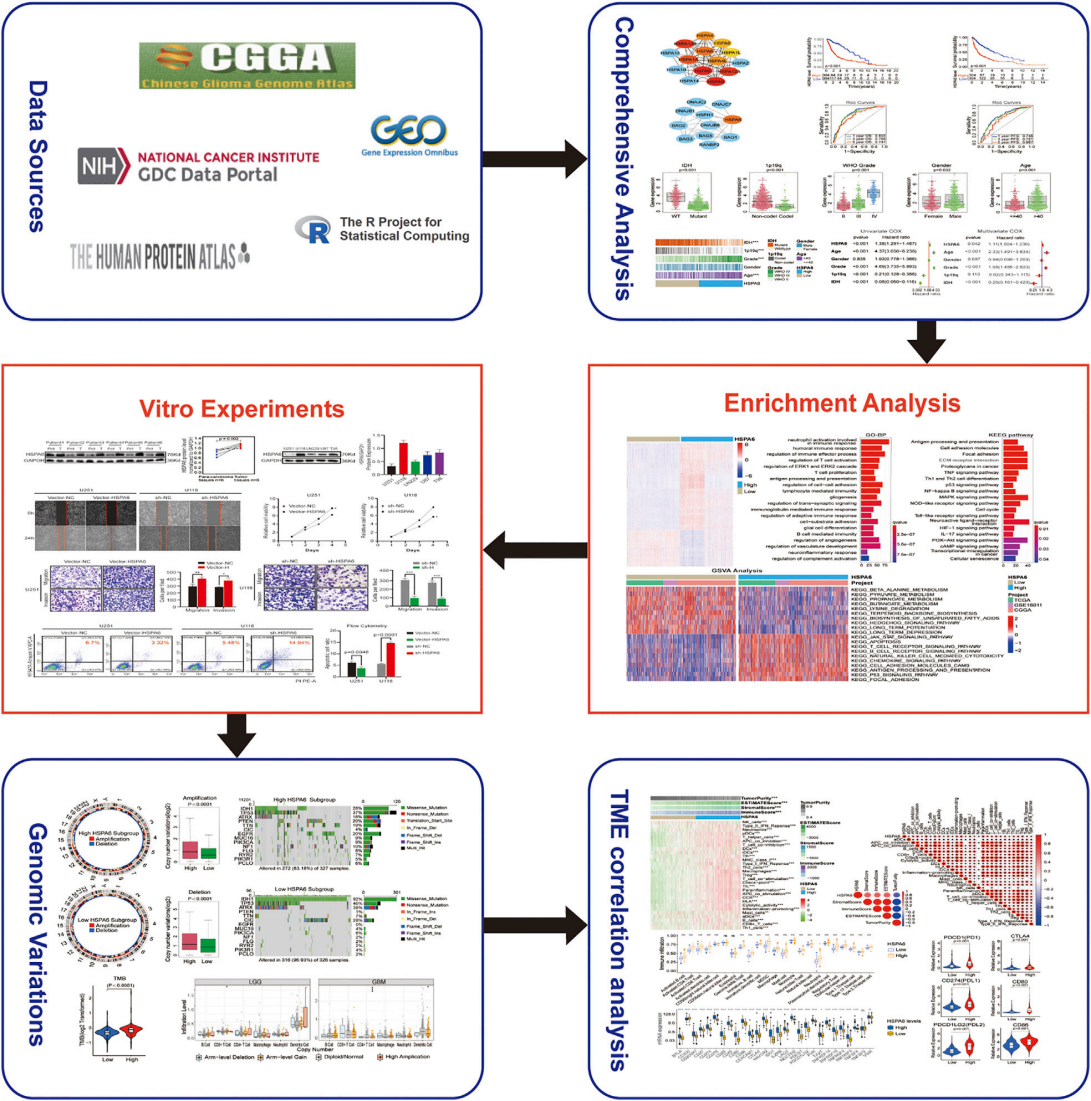
Flow diagram illustrating the study design.

Figure 1 depicts a flow diagram that describes the overall research process.

### Dataset Acquisition and Pre-Processing

We downloaded all databases featuring patients with gliomas from the TCGA (https://portal.gdc.cancer.gov/) and CGGA (www.cgga.org.cn/) databases. We also downloaded information from a microarray dataset (GSE16011) from the Gene Expression Omnibus (GEO; www.ncbi.nlm.nih.gov/geo/) database. The inclusion criteria for glioma samples were as follows: 1) patients with WHO grade II, III, or IV glioma, 2) patients diagnosed with glioma with OS of more than 30 days, 3) patients with expression data, and 4) patients with primary glioma. After filtering, we acquired 1810 glioma samples (TCGA, *n* = 608; CGGA, *n* = 965; GSE16011, *n* = 237). The clinical classifications for the 1810 patients with glioma are shown in Supplementary Table S1. We transformed the transcripts per kilobase million (TPM) values from RNA-sequencing data, and robust multichip averaging analysis (RMA)-processed values from the GSE16011, by log2 to allow easier comparison. In addition, somatic mutations and frequency data were processed by the maftools package within R (www.r-project.org/). The GISTIC algorithm was taken to process amplification and deletion of copy number variation (CNV) data (Mermel et al., 2011). In total, 29 immune-associated gene sets, and 28 key ICPs, as based on the methods described by Auslander (Auslander et al., 2018).

Six paired frozen specimens, along with para-carcinoma tissues, were acquired from surgical patients with gliomas at The Second Affiliated Hospital of Nanchang University between 2016 and 2021.

### Protein Network Construction

We generated a protein-protein interaction (PPI) network for the HSP superfamily; this network featured 182 members (Supplementary Table S2). Next, we analyzed the HSP70 family, and HSPA6, by applying STRING version 11.5 and Cytoscape software (Otasek et al., 2019). To further investigate the core role of HSPA6 within the HSP70 family, we calculated the top 10 networks through using CytoHubba tool in Cytoscape; these were ranked by the MCC method.

### Clinicopathological Characteristics

On the grounds of the median expression level of HSPA6 in the three cohorts, patients with gliomas were separated into groups exhibiting low and high of HSPA6 expression levels. The Kaplan-Meier method was used to perform survival analysis and evaluate the outcomes of patients with gliomas in both the low and high expression subgroups; two parameters were generated: overall survival (OS) and progression-free survival (PFS; only for the TCGA cohort). ROC curves and AUC values were utilized to evaluate the predictive accuracy of HSPA6 expression across the three cohorts. Next, we exploited the survival package in R to perform univariate Cox regression analysis of five clinical factors (age, gender, grade, 1p19q co-deletion, IDH mutations), and HSPA6 expression, to identify key factors related to survival. Meanwhile, multivariate Cox regression analysis was utilized to differentiate the independent factors of prognosis.

### Functional Enrichment Analysis

The limma package in R was utilized to distinguish differentially expressed genes (DEGs) between the glioma patients showing low and high expression levels of HSPA6 expression; DEGs were defined by a log2 (fold change) >1 and *p* < 0.05 (Ritchie et al., 2015). GO-BP and KEGG enrichment analyses (based on *p* < 0.05 and *q* < 0.05) were then carried out on the DEGs using the clusterProfiler, enrichplot, and ggplot2 packages in R (Yu et al., 2012). In order to identify significantly enriched pathways in the patients’ subgroups showing low and high of HSPA6 expression, we performed GSVA in the R environment, incorporating—data from c2.cp. kegg. v6.2. symbols in the Molecular Signatures Database (MSigDB) database (www.gsea-msigdb.org/gsea/msigdb/) (Hänzelmann et al., 2013). KEGG analysis identified significant pathways between the two groups of patients on the basis of an adjusted *p* < 0.05, a log2 (fold change) >0.1, and a false discovery rate (FDR) < 0.05.

### Ethical Approval

The research protocol was approved by the medical ethics committee of the Second Affiliated Hospital of Nanchang University. We got written informed consent from all the patients with glioma.

### Cell Culture and Plasmid Transfection

Several human glioma cell lines (U251, U87, U118, LN229, and T98) were purchased from the Shanghai Institute of Biosciences and Cell Resources Center (Shanghai, China). These cell lines were cultured with Dulbecco’s modified Eagle medium (DMEM, ATCC) and 10% fetal bovine serum (Gibco, United States) at 37°C and 5% CO_2_. When the cell density reached 70–90%, we performed transfection using Lipofectamine 3000 (Thermo Fisher, L3000075, United States). Cells were then separated into an overexpressed group, a knockdown transfection group, a negative control (NC) group, and an untreated group. The primers used to create the HSPA6 overexpression plasmid were as follows: forward, 5′-TAC​CGG​ACT​CAG​ATC​TCG​AGC​GCC​ACC​ATG​CAG​GCC​CCA​CGG​GAG​CTC-3′; reverse, 5′-GAT​CCC​GGG​CCC​GCG​GTA​CCG​TAT​CAA​CCT​CCT​CAA​TGA​TGG​GGC-3′. The HSPA6 siRNA sequence was: 5′-GCC​CGC​CTA​TTT​CAA​TGA​CTC-3′.

### Western Blotting

Proteins from cells and tissues lysates were extracted with radioimmunoprecipitation (RIP) assay buffer (Solarbio, Beijing, China) containing proteinase inhibitors. The primary antibodies were HSPA6 (1:1000, DF8465 affinity Biosciences Ltd., China), the PI3K (1:2000, 67071-1-Ig, Proteintech, China), AKT1 (1:1000, 10176-2-AP, Proteintech), pAKT1-S473 (1:2000, 66444-1-Ig, Proteintech), pAKT1-T308 (1:1000, #4056S, Cell Signaling Technology, United States), and glyceraldehyde-3-phosphate dehydrogenase (GAPDH) (1:20000, 60004-1-lg, Proteintech, Wuhan, China). Then, 10% sodium dodecyl sulfate polyacrylamide gel electrophoresis (SDS-PAGE) was utilized to separate the proteins which were then transferred onto a 0.22 μm PVDF membrane (Millipore, MA, United States). Next, the membranes were blocked with 5% skimmed milk. Subsequently, the membranes were incubated with primary antibodies overnight at 4°C followed by appropriate secondary antibodies (anti-rabbit antibody, 1:5000, SA00001-2; anti-mouse antibody, 1:5000, SA00001-1; Proteintech, Wuhan, China). Finally, the membranes were incubated with enhanced chemiluminescence (ECL) substrate (Thermo Fisher Scientific, MA, United States) and developed with GV6000M imaging system (GelView 6000pro).

### Quantitative Real-Time PCR and RNA-seq

Total RNA was extracted from cells with the Simply P Total RNA Extraction Kit (Bioflux, Beijing, China) and reverse-transcribed to synthesize cDNA with HiScript III-RT SuperMix for qPCR (Vazyme, Nanjing, China). The ChamQ Universal SYBR qPCR Master Mix (Vazyme, Nanjing, China) was used for qPCR. The primer sequences were as follows: the forward HSPA6 primer was 5′-CGT​GCC​CGC​CTA​TTT​CAA​TG-3′, the reverse HSPA6 primer was 5′-AAA​AAT​GAG​CAC​GTT​GCG​CT-3′; the forward GAPDH primer was 5′- GAA​CGG​GAA​GCT​CAC​TGG-3′, and the reverse GAPDH primer was 5′- GCC​TGC​TTC​ACC​ACC​TTC​T-3′. RNA samples were sequenced and analyzed by Shanghai Majorbio Biotechnology Co., Ltd. and Shanghai Jiayin Biotechnology Co.; Ltd. RNA-seq transcriptome library was prepared following TruSeqTM RNA sample preparation Kit from Illumina (San Diego, CA, United States) using 1 μg of total RNA.

### Wound‐Healing Assay

5 × 10^5^ cells were cultivated per well on six‐well plates using the medium described above; the plates were covered and incubated overnight. The following morning, the cells were scratched in the middle of the cell plate with the tip of a 200 μl sterile spear to form artificial wounds. After washing with phosphate‐buffered saline, the medium was replaced with a serum-free medium. At 0 and 24 h, images of the wound‐healing process were photographed with an inverted Leica microscope with a ×10 objective lens. The width of the scratch edge was compared at different time points.

### Cell Proliferation Assays

Cell Counting Kit-8 (CCK-8) assays were used to detect cell proliferation. In total, 5 × 10^3^ viable cells were cultivated per well on 96‐well plates in a final volume of 100 μl DMEM containing 10% FBS. After 1–4 days of incubation, 10 μl of CCK8 (Beyotime, C0037, China) was added and incubated for 4 h. Then, the absorbance values of the plates were measured at a wavelength of 450 nm.

### Transwell Migration and Invasion Assays

Transwell chambers (Corning, United States) were used to execute migration and invasion assays. First, the chambers were covered with 500 μg/ml of Matrigel (Corning, 356234 United States). Then, 5 × 10^5^ cells were added to each well and cultured with 200 μl of serum-free medium. Then, the lower chamber of the Transwell was covered with 600 μl of DEME with 10% fetal bovine serum. After 24 h of incubation, the non-invasive cells on the upper surface of the chambers were wiped away with a cotton swab. The chambers were then fixed with 4% paraformaldehyde (Solarbio, P1110, China) for 30 min, dyed with Crystal Violet stain (Solarbio, G1075, China) 30 min, and then imaged with a Leica Microsystems D-35578 microscope (five random 200 × fields per well). The migration assay was performed in the same ways as the invasion assay, except that the bottom of the chamber did not contain Matrigel.

### Flow Cytometry

A YF 647A-Annexin V and PI Apoptosis Kit (Yuheng Biotechnology Co., Ltd., Suzhou, China) was used for flow cytometry. First, the medium was gathered in a 15 ml centrifuge tube. Then, the cells were digested in trypsin-free EDTA, centrifuged with the retained medium at 300 g for 5 min at 4°C, and the supernatant was discarded. Next, the cells were resuspended and centrifuged twice with PBS under the same conditions. The supernatant was then got rid of and the cells were gently resuspended in 100 μl of binding buffer. Then, 5 μl of YF647A-Annexin V and 5 μl of PI were mixed to each tube and incubated at 24°C in the dark for 15 min. At last, the experiment was detected with a CytoFLEX LX system (Beckman Coulter, United States); data were analyzed with CytExpert software.

### A Comparison of CNV and TMB Between Patients With Low and High Expression Levels of HSPA6

The RCircos tool was used to identify and visualize meaningful genomic amplifications and deletions (Zhang et al., 2013). Maftools and GenVisR were used to calculate and compare mutation categories, and the frequencies of mutant genes, between groups of patients with low and high expression levels of HSPA6 (Skidmore et al., 2016; Mayakonda et al., 2018). Considering that this is a newly developing biomarker for immunotherapy, we compared the tumor mutational burden (TMB) between the two different subgroups of HSPA6 expression. In addition, the correlations between CNV for HSPA6 and six TIICs were investigated employing the Tumor Immune Estimation Resource (TIMER).

### Single Sample Gene Set Enrichment Analysis

The enrichment of 29 TIICs in the tumor microenvironment (TME) was quantitatively analyzed by single sample gene set enrichment analysis (ssGSEA). To further detect the association between HSPA6, TME, and TIICs, we used the corrplot package. The ssGSEA algorithm was then exploited to estimate the abundance of each TIIC.

### Immune Checkpoint Analysis

We identified 28 immune checkpoints (ICPs) from the existing literature (Auslander et al., 2018). To investigate the correlations between the expression of HSPA6 and these 28 ICPs, we used Sanger box (http://sangerbox.com/Tool). In addition, we investigated the correlation between paired immune checkpoints (PD1, CD274, PDL2; CTLA4, CD80, and CD86) and HSPA6 expression.

### Statistical Analysis

Comparisons between two groups were calculated by the student’s t-test. Comparisons between three or more groups were computed by the Kruskal–Wallis test. Clinicopathological correlation analysis was performed with Pearson’s Chi-squared test. Kaplan-Meier analysis and Cox regression were performed for univariate and multivariate prognosis analyses, respectively. Two-sided tests were used for all statistical analyses and all analyses were carried out with GraphPad Prism 8 (GraphPad Software, Inc.), SPSS Statistics, version 25 (IBM, United States), and R programming, version 4.0.3. *p* < 0.05 was regarded as being statistical significance. All the vitro experiments were performed independently and in triplicate.

## Results

### HSPA6 may be a Poor Prognostic Biomarker for Patients With Glioma

**FIGURE 2:**
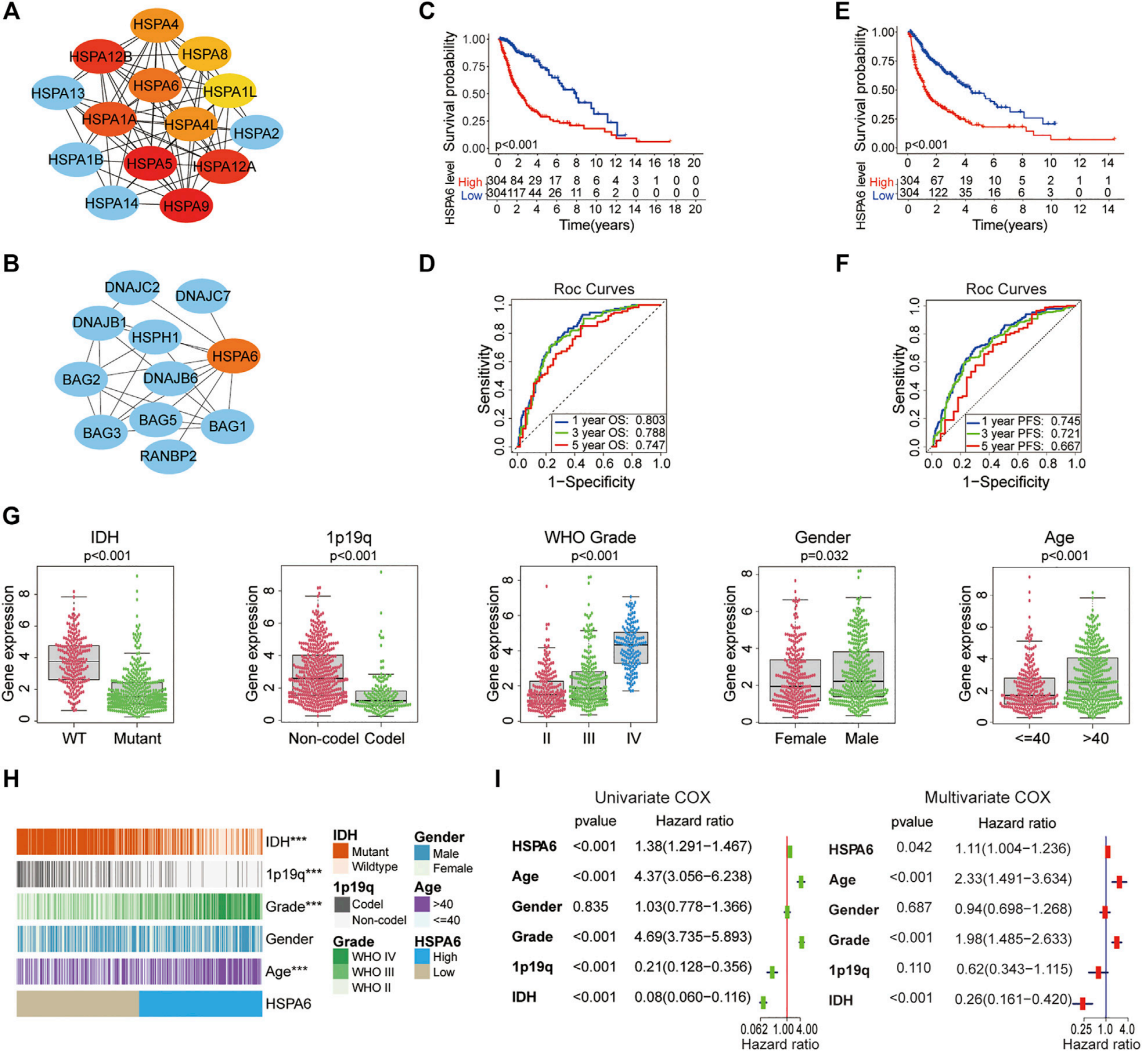
HSPA6 may be a poor prognostic and predictive biomarker for patients with glioma. **(A)** A core gene map for the HSP70 family network was constructed by using Cytoscape. Increasing red coloration indicates a stronger core. **(B)** A PPI network for HSPA6 was constructed by Cytoscape. **(C,E)** Kaplan‒Meier overall survival (OS) and progression free survival (PFS) curves for glioma patients in accordance with HSPA6 expression in The Cancer Genome Atlas (TCGA) cohort. **(D,F)** Time-dependent receiver operating characteristic curves for the prognostic model in the TCGA cohort (for predicting 1-, 3-, and 5-year overall OS and PFS). **(G)** Variance analysis of HSPA6 expression with regards to various clinical traits [IDH, 1p/19q, grade, gender and age] in the TCGA cohort. **(H)** Relationship between HSPA6 expression and the clinical features of gliomas. ***p < 0.001. **(I)** Univariate and multivariate cox regression analyses based on clinical features (HSPA6, age, gender, grade, 1p/19q and IDH) in the TCGA cohort (*p* < 0.05). Risk factor: hazard ratio (HR)>1; protective factor: HR < 1.

A total of 182 members of the HSP superfamily exist in the human genome. We employed Cytoscape to construct a PPI network for members of the HSP superfamily and found that HSPA6 is directly or indirectly associated with 47 family proteins (Supplementary Figure S1A). According to the correlation score (Supplementary Table S3), the core network map showed that the core function of HSPA6 protein ranked sixth in the HSP70 family (Figure 2A). We also found several chaperone proteins of the BAG family that were associated with HSPA6 (Figure 2B). Patients with gliomas were split into low and high subgroups based on their median expression level of HSPA6 in the TCGA. Survival analyses showed that HSPA6 may be a poor prognostic biomarker for OS and PFS (Figures 2C,E). Similar trends were identified for the CGGA and GSE16011 cohorts (Supplementary Figures S1B,D). Furthermore, the AUCs for 1-, 3-, and 5-year OS were 0.803, 0.788, and 0.747, respectively; while the AUCs for 1-, 3-, and 5-year PFS were 0.745, 0.721 and 0.667, respectively (*p* < 0.01 for all) (Figures 2D,F). In the CGGA cohort, the AUCs for 1-, 3-, and 5-year OS were 0.714, 0.765, and 0.768, respectively. In the GSE16011 dataset, the AUCs for 1-, 3-, and 5-year OS were 0.751, 0.817, and 0.738 (Supplementary Figures S1C,E).

Therefore, the correlation between HSPA6 expression and clinical factors (IDH, 1p/19q, grade, gender, and age) were investigated in the three cohorts. As shown in Figures 2G,H, in the TCGA cohort, the clinical traits differed significantly between patients with high levels of HSPA6 expression and patients with low levels of HSPA6 expression (*p* < 0.001), except for gender in the TCGA cohort. Similar presentations were also obtained in the CGGA and GSE16011 cohorts (Supplementary Figures S2A,B,E,F).

Moreover, Univariate and multivariate Cox regression analyses displayed that HSPA6 was meaningfully statistical significance (*p* < 0.05) which was associated with clinical outcome in patients with gliomas in the three cohorts (Figure 2I and Supplementary Figures S2C,F). Collectively, these results suggested that HSPA6 may be an independent but poor prognostic biomarker for patients with glioma.

### Function Annotation of HSPA6-Related Genes

**FIGURE 3:**
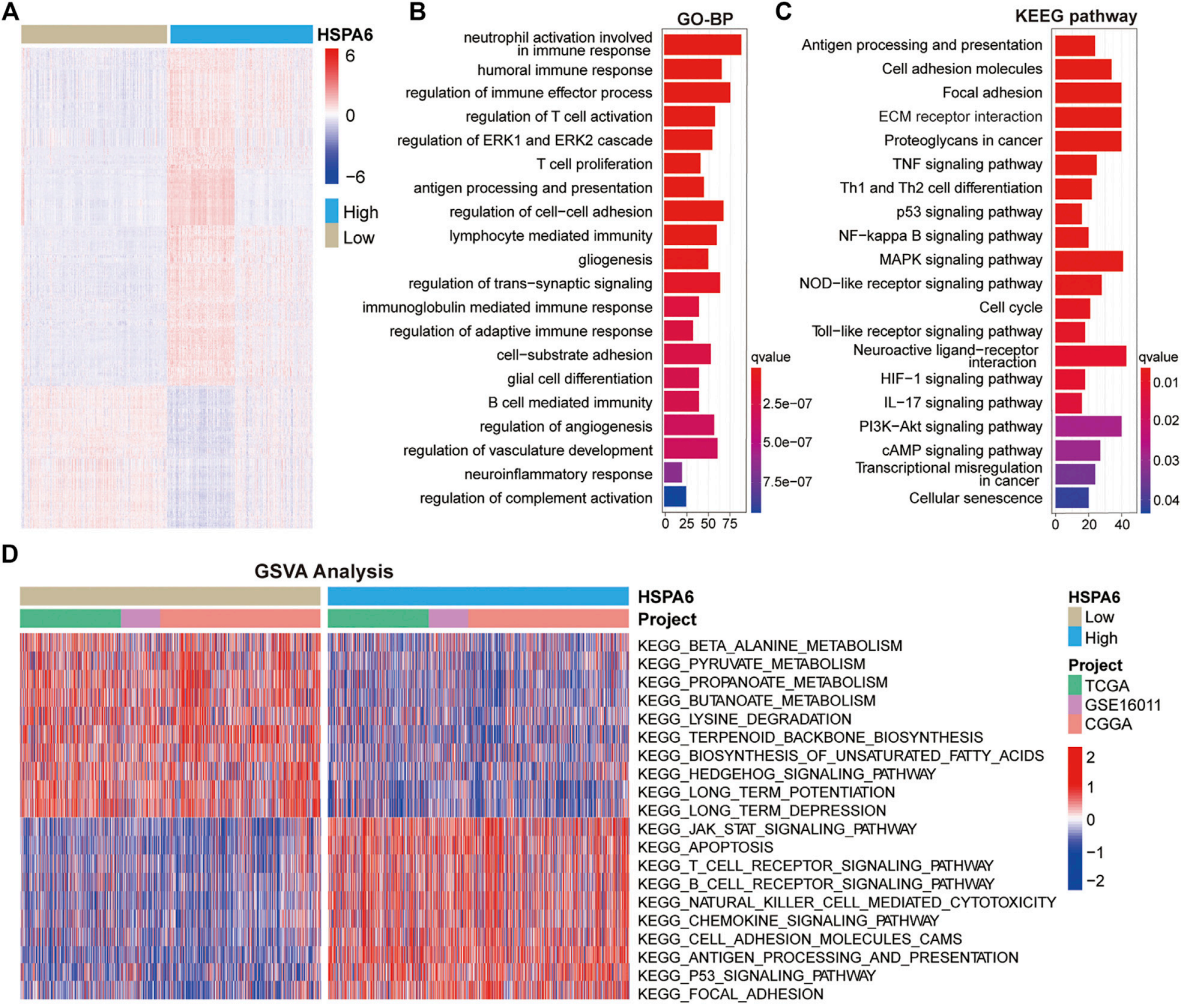
Functional annotation of subgroups with high and low expression levels of HSPA6. **(A)** Differentially expressed genes (DEGs) between the subgroups of patients with low and high HSPA6 expression were identified by the Wilcoxon rank sum test in the TCGA cohort (FDR < 0.05 and log2Fold Change >1) cohort. **(B,C)** GO (Gene Ontology) and KEGG (Kyoto Encyclopedia of Genes and Genomes) enrichment analysis for DEGs (*p* < 0.05 and q < 0.05). There were 1669 DEGs in the TCGA cohort. **(D)** GSVA (Gene Set Variation Analysis) in the TCGA cohort, Chinese Glioma Genome Atlas (CGGA) cohort, and GSE16011 cohort, as determined by Gene Expression Omnibus (red = high score, blue = low score).

To further investigate the mechanisms undergoing how HSPA6 may be associated with the outcome of patients with glioma, we identified DEGs by comparing subgroups with low and high expression levels of HSPA6 in the three cohorts (FDR <0.05 and log2Fold Change >1) using the Wilcoxon rank sum test. We identified 1669 DEGs in the TCGA cohort (Supplementary Table S4), 1114 in the CGGA cohort (Supplementary Table S5), and 581 in the GSE16011 cohort (Supplementary Table S6). All significant DEGs are shown in the heatmap in Figure 3A. GO enrichment analysis showed that these DEGs were closely associated with immune regulation, cell adhesion, and angiogenesis (Figure 3B). Similarly, KEGG enrichment analysis identified pathways associated with immune pathways, adhesion, and tumorigeneses, such as antigen processing and presentation, ECM receptor interaction, and the p53 signaling pathway (Figure 3C). Similar functional annotations were also verified in the TCGA and GSE16011 cohorts (Supplementary Figures S3A,B).

In addition, we conducted GSVA to investigate the potential pathways associated with the low and high subgroups of HSPA6 expression in patients with gliomas. When comparing the three cohorts, we found that the subgroup with low expression levels of HSPA6 was closely related with tumor metabolism-related mechanisms, such as beta-alanine, pyruvate and propanoate metabolism. The subgroup with high expression levels was associated mainly with the development of tumors, cancer-related signaling pathways, and immune responses, such as apoptosis and focal adhesion, antigen processing and presentation, and the Jak-Stat and P53-signaling pathways (Figure 3D). These results indicated that HSPA6 is closely related to immune regulation in patients with glioma.

### *In Vitro* Investigations of HSPA6

**FIGURE 4:**
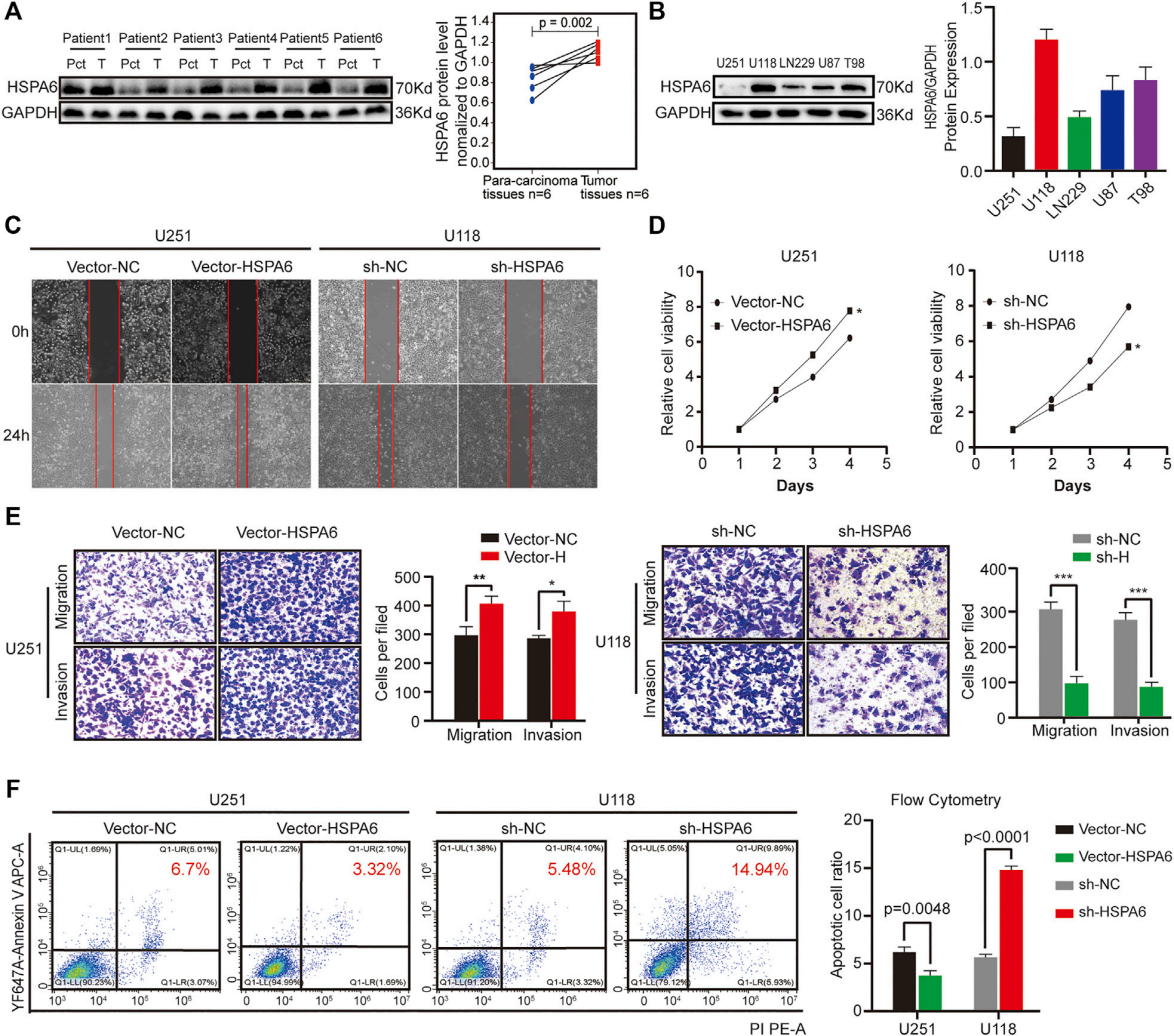
Expression verification of HSPA6 in clinical brain tissues and different glioma cell lines and experimental validation of HSPA6 in U251 and U118 cells. **(A)** Western blot showing HSPA6 expression in six paired glioma tissues and matched para-carcinoma tissues from the same patient. HSPA6 protein expression levels were quantified by ImageJ software. **(B)** Western blot of HSPA6 expression in different glioma cell lines. HSPA6 protein expression levels were quantified by ImageJ software. **(C)** Cell scratch test to detect the migration ability of U251 and U118 cells which had been transfected with Vector-NC or Vector-HSPA6 and the sh-NC or sh-HSPA6 plasmids. The width of the wound was photographed under a microscope (magnification, 100x). **(D)** Cell Counting Kit-8 (CCK-8) assays were used to detect the proliferation of U251 and U118 cells which had been transfected with Vector-NC or Vector-HSPA6 and the sh-NC or sh-HSPA6 plasmids. **(E)** Transwell assays of U251 and U118 cells which had been transfected with Vector-NC or Vector-HSPA6 and sh-NC or sh-HSPA6 plasmids. Representative photographs (magnification, 200×) of migratory or invading cells on the membrane coated with or without Matrigel. Quantitative analysis of Transwell assays was performed by ImageJ software. **(F)** Flow cytometry detection of apoptosis in U251 and U118 cells which had been transfected with Vector-NC or Vector-HSPA6 and sh-NC or sh-HSPA6 plasmids. The proportion of apoptotic cells was equal to the sum of the proportion of early and late apoptosis. Data are expressed as the mean ± SEM from three independent experiments, *p < 0.05, **p < 0.01, ***p < 0.001.

We acquired glioma samples from six patients undergoing surgery in the Second Affiliated Hospital of Nanchang University. The expression levels of HSPA6 proteins were much higher in the glioma tissues compared with matched para-cancerous tissue. (Figure 4A). To further verify the association between HSPA6 expression and the malignant progression of glioma, we inspected the differential expression of HSPA6 in these glioma cell lines. We found that HSPA6 expression was the lowest in U251 cells and the highest in U118 cells (Figure 4B). To further investigate the function of HSPA6 in U251 and U118 cells, we constructed plasmids to overexpress and knockdown HSPA6 in U251 and U118 cells, respectively. Q-PCR and western blotting (WB) confirmed that HSPA6 expression in U251 cells transfected with the overexpression plasmid was significantly higher than in the control and untreated cell groups (*p* < 0.05, Supplementary Figures S4A,B). The same methods also proved that HSPA6 expression in U118 cells transfected with the knock-down plasmid was significantly lower than in the control and untreated cell groups (*p* < 0.05, Supplementary Figures S4C,D).

Next, a range of functional experiments were undertaken to investigate the connection between HSPA6 expression and the degree of malignancy in glioma cells from different treatment groups. Wound healing assays revealed that the migration capacity of the U251 Vector-HSPA6 group was significantly enhanced and the ability of the U118 sh-HSPA6 group was significantly reduced when compared to the control group (Figure 4C). CCK-8 assays further showed that the proliferative capacity of the U251 Vector-HSPA6 group was significantly promoted while the proliferative capacity of the U118 sh-HSPA6 group was significantly reduced when compared to the control group (*p* < 0.05, Figure 4D). Transwell assays showed that those capacities of the U251 Vector-HSPA6 group were significantly improved and that the migration and invasion capacities of the U118 sh-HSPA6 group were significantly reduced when compared to the control group (*p < 0.05, **p < 0.01, ***p < 0.001, Figure 4E). Flow cytometry analyses also disclosed the apoptotic cell ratio of the U251 Vector-HSPA6 group was significantly declined and that the apoptotic cell ratio of the U118 sh-HSPA6 group was significantly ascended when compared to the control group (*p* < 0.005, Figure 4F).

In order to further study the related pathway mechanism of HSPA6 in glioma cell lines, the differential gene pathway enrichment was analyzed by RNA sequencing after HSPA6 overexpression in the U251 cell line. The result also indicated that HSPA6 was closely related to tumor cell immunity, proliferation, and apoptosis, such as antigen processing and presentation and PI3K-AKT signaling pathways (only top ten, Supplementary Figure S4E). It is often known that PI3K activates or inhibits a series of downstream substrates such as apoptosis-related proteins (Bad and Caspase9) after phosphorylation of AKT, thus regulating cell proliferation, differentiation, apoptosis, migration, and other phenotypes. Western blotting confirmed that the expression of PI3K and pAKT (Ser473 and Thr308) in the HSPA6 overexpressed U251 cell group were significantly higher than those in the control group, while the total protein expression of AKT basically changed little (Supplementary Figure S4F). Collectively, these results indicated that HSPA6 may influence the degree of malignancy in glioma cells by interacting with PI3K-AKT signaling pathway.

### Differences in Genomic Variation Between the Two Subgroups

**FIGURE 5:**
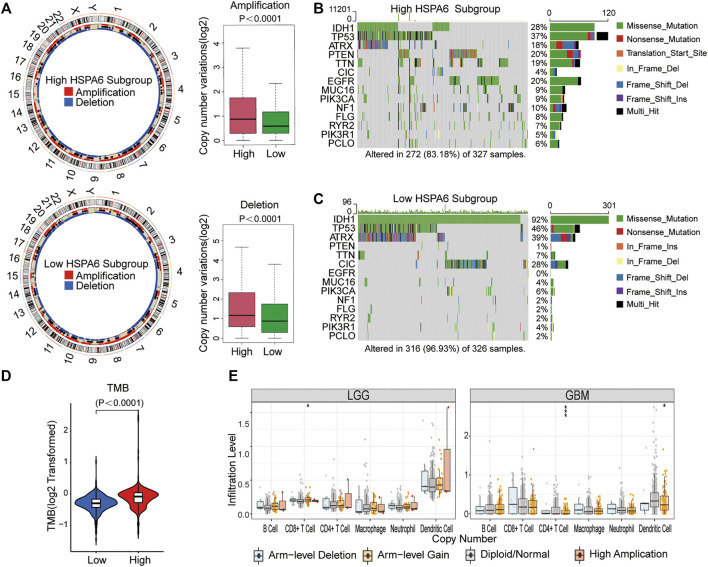
Genomic variations of subgroups with low and high HSPA6 expression and associations between HSPA expression and tumor-infiltrating immune cells (TIICs). **(A)** Left: Circos plots illustrating amplification and deletion in the two subgroups. Right: A significant difference was detected in copy number variation (CNV) frequencies between two subgroups. **(B,C)** Waterfall plots illustrating the somatic mutations of 14 genes in tumors from the two subgroups. **(D)** Differential tumor mutational burden (TMB) between the two subgroups. **(E)** Associations of HSPA6 CNV with six types of TIICs. *p < 0.05; ***p < 0.001.

In view of the obligatory function of genomic variation in immune cell infiltration and tumor immunoregulation, CNV and somatic mutation were used to identify distinguishing genomic alterations in the subgroups showing differential HSPA6 expression. Both the amplification and deletion frequency of CNV was significantly higher in the subgroup with high expression levels when compared to the subgroup with low expression levels. (Figure 5A). Next, we created a “waterfall” map of somatic mutations; this revealed that each subgroup possessed genes with different mutations. The proportions of patients in the subgroup with high expression levels of HSPA6 with mutations in IDH1 (28%), ATRX (18%), PTEN (20%), TTN (19%), and EGFR (20%) was significantly different from those in the subgroup with low expression levels of HSPA6. (*p* < 0.01), whereas the frequency of TP53 was similar between the two subgroups. (Figures 5B,C). In addition, the subgroup with high expression levels of HSPA6 tended to have a significantly higher TMB (*p* < 0.0001, Figure 5D). Finally, three of the six types of TIIC were found to be associated with HSPA6 CNV in patients with low grade gliomas (LGGs) and glioblastoma (GBM) (Figure 5E). Collectively, these results implied that patients with gliomas with high expression levels of HSPA6 exhibited a special immunological reaction.

### The Role of HSPA6 in Immune Cell Infiltration and Potential Responses to Immunotherapy

**FIGURE 6:**
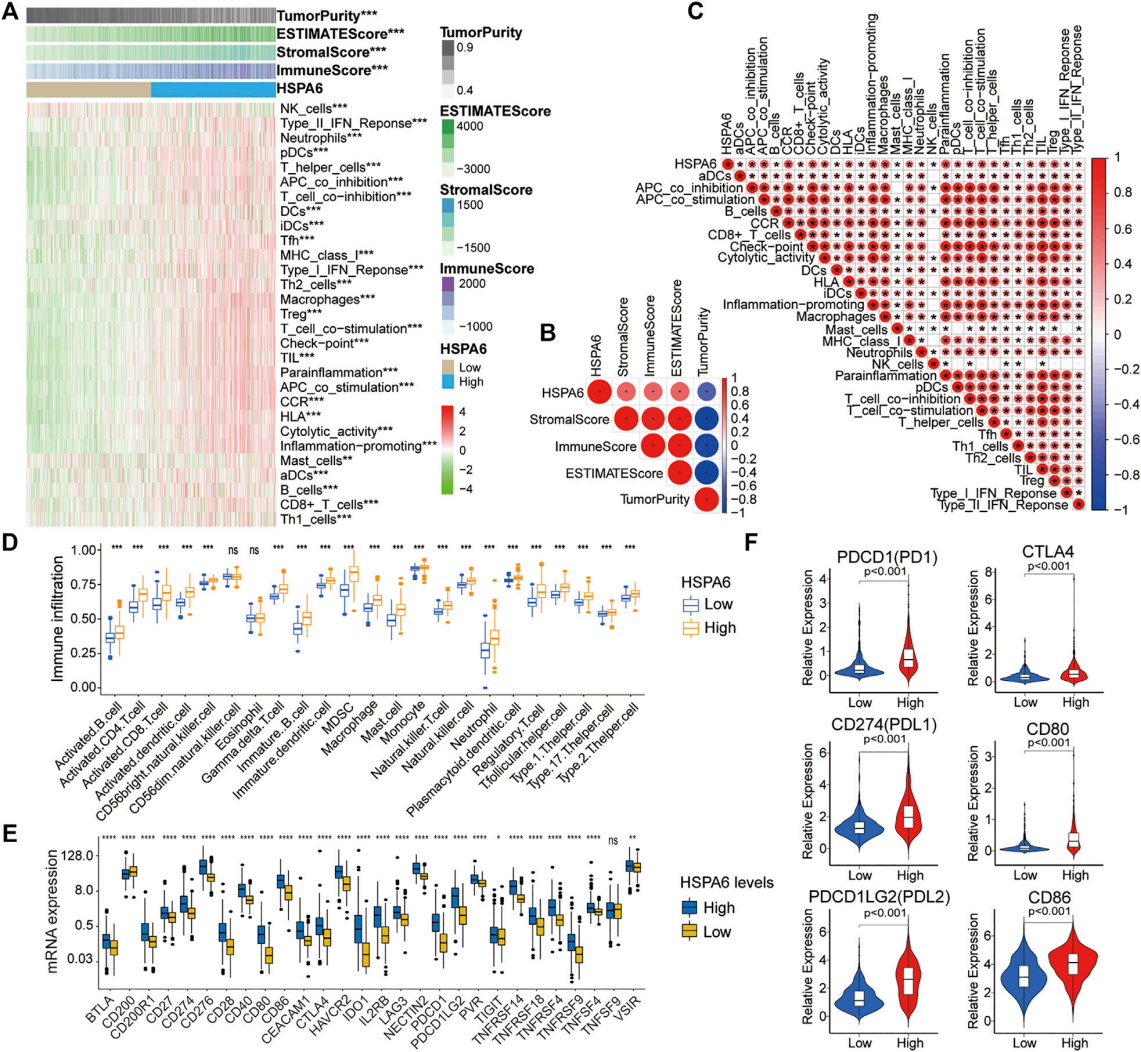
Different tumor microenvironment (TME) characteristics of the two subgroups in the TCGA cohort. **(A–C)** Correlations of HSPA6 expression with 29 immune-associated gene sets, immune score, stromal score, ESTIMATE score, and tumor purity. **(D)** Different abundances of TIICs when compared between subgroups of patients with low and high expression levels of HSPA6. **(E)** Differential analysis of several immune checkpoint (ICP) expression levels between patients with low and high HSPA6 expression levels. **(F)** Differential expression of different immune checkpoint receptors and ligands between the two subgroups. Correlation analysis was performed by Pearson’s correlation analysis. ns, *p* > 0.05, *p < 0.05, **p < 0.01, ***p < 0.001, ****p < 0.0001.

Significant correlations of HSPA6 expression with 29 immune-associated signatures and immune correlation scores, were determined for the TCGA cohort (**p < 0.01, ***p < 0.001, Figures 6A,C). HSPA6 expression was positively associated with immune score, stromal score, and ESTIMATE score, but negatively correlated with tumor purity (Figure 6B). To investigate the correlation between immune infiltration and HSPA6 expression, we used the ssGSEA algorithm to determine the enrichment of the 23 signatures with immune. There was a significantly greater number of immune-related signatures (***p < 0.001, Figure 6D) in the subgroup of patients with high HSPA6 expression levels than in the subgroup with low expression levels. Next, we investigated the differences in expression of diverse immune checkpoints between the subgroups of glioma patients with low and high HSPA6 expression. We found that the majority of immune checkpoints differed significantly between the two subgroups (Figure 6E). Similar results were also acquired from the CGGA and GSE16011 cohorts (Supplementary Figures S5, S6). Figure 6F shows that the relative expression levels of immune checkpoint receptors and ligands in the subgroup of patients with high expression levels of HSPA6 were significantly higher than those in the subgroup with low expression levels (*p* < 0.001). These results imply that HSPA6 may be closely related to the immune microenvironment.

## Discussion

Gliomas are intracranial neoplasms that stem from neuroglial progenitor cells (Cheng et al., 2020). Despite different combinations of surgery, temozolomide targeted-chemotherapy, and radiotherapy, patients with glioma still have a poor quality of life and a short survival time, especially those with high-grade gliomas (Touat et al., 2017). Traditional treatments are often ineffective for patients with glioma; consequently, there is a clear need to identify effective prognostic and therapeutic agents for patients with gliomas. In our study, we explored the specific value of HSPA6 in glioma.

First, we constructed a PPI network for members of the HSP superfamily; this showed that HSPA6 is directly or indirectly associated with 47 proteins in the HSP family. This also showed that HSPA6 had complex and extensive connections with other members of the HSP superfamily. This network also identified proteins that are directly related to HSPA6, including those in the HSP superfamily, and others in the BCL-2 associated athanogene (BAG) family, including BAG1, BAG2, BAG3, and BAG5. One previous study reported that the modular structure of the BAG family members enables interaction with a variety of proteins to exert pro-tumorigenic roles (Mariotto et al., 2020). Analysis of OS and PFS analyses indicated that the subgroup of patients with high expression levels of HSPA6 had a poor prognosis when compared with patients with low expression levels of HSPA6 in the TCGA cohort. ROC curve analyses were conducted to calculate the predictive accuracy of the prognostic model in patients with gliomas. This led to the question as to what controls the expression of HSPA6 protein in patients with glioma. The association between HSPA6 expression and relevant clinical factors in patients with glioma further indicated that there were significant differences in certain clinical traits, including IDH, 1p/19q, grade, and age. Moreover, univariate and multivariate Cox regression analysis showed that HSPA6 may represent a poor but independent prognostic biomarker for glioma. These results were further verified in both the CGGA and GSE16011 cohorts.

GO and KEGG analysis of DEGs showed that HSPA6 expression was closely associated with immune regulation, cell adhesion, and angiogenesis across all three cohorts. In addition, GSVA analysis revealed that the subgroup of patients with low expression levels of HSPA6 were mainly associated with metabolism-related mechanisms, while the subgroup of patients with high expression levels of HSPA6 were mainly associated with cancer-related signaling pathways and immune responses. These results were also in line with the cancer-promoting mechanism of HSP70s, including the regulation of apoptosis pathways, tumor immunity, and the induction of angiogenesis (Albakova et al., 2020).

In recent years, there are more and more studies on HSPA6 protein in different tumors. However, the signaling pathway and mechanism of HSPA6 are still unclear in the field of cancer research. Shin et al. (Shin et al., 2017) found that HSPA6 induces phosphorylation of MAPK and AKT signals in bladder cancer cells and inhibits transcription factor related MMP-9 regulation to act on garlic extract, thus enhancing its mediated inhibition of bladder cancer cell proliferation, migration, and invasion; Meanwhile, Wang et al. (Wang et al., 2020b) showed that the expression of ARHGEF10L can stimulate gastric cancer by promoting RhoA-Rock1-phosphorylation-ERM signal transduction, inducing epithelial-mesenchymal cell transformation (EMT) and increasing HSPA6 expression.

We also verified that HSPA6 protein was expressed at significantly higher levels in clinical glioma specimens than in matched para-cancerous tissues. Next, we further verified the relationship between HSPA6 and the malignant progression of glioma cell lines *in vitro*. Interestingly, we found that the overexpression of HSPA6 accelerated the migration, proliferation, and invasion abilities of glioma cells. However, the opposite effect was observed following the knockdown of HSPA6 in glioma cells. The PI3K-AKT pathway is a critical regulatory pathway for cell proliferation and metabolism and is closely related to tumorigenesis and development (Janku et al., 2018). When receptor tyrosine kinases and G protein-coupled receptors are activated, PI3K phosphorylates phosphatidylinositol 3,4-diphosphate to produce 3,4,5-triphosphate phosphatidylinositol (PIP3). Activated PIP3 recruits and activates AKT in the cytoplasm, which in turn affects the level of related transcription factors involved in EMT to promote cell invasion and metastasis (Wu et al., 2015). In our study, we preliminarily explored that HSPA6 may affect the malignant degree of glioma cells through interaction with the PI3K-AKT signaling pathway. These results manifested that HSPA6 may be a potentially reliable therapeutic target for patients with glioma.

A growing body of research indicates that the TME is essential for the growth and aggressive nature of gliomas (Chang et al., 2016; Qian et al., 2018; Meng et al., 2019). Over the past few years, immunotherapeutic strategies targeting ICPs has achieved promising results in multiple forms of human cancer (Boussiotis and Charest, 2018; Sun et al., 2020b). Genomic alterations can predict disease classification and the prognosis of patients with gliomas (McNulty et al., 2019). Both amplification and deletion showed that the frequency of variants in the subgroup with high HSPA6 expression levels was significantly higher than that in the subgroup with low expression levels. Furthermore, the frequency of several types of common somatic gene mutations varied in the subgroups showing low and high expression levels of HSPA6. The loss of PTEN is known to lead to metabolic abnormalities in the lipid product of PI-Kinase (PIP3), thus directly antagonizing the activation of the carcinogenic PI3K/AKT/mTOR signaling network (Álvarez-Garcia et al., 2019). When applied for clinical targeted therapy, the EGFR is usually mutated in patients with glioma (Oh et al., 2021). EGFR has been shown to be implicated in tumorigenesis as an oncogene. EGFR signaling leads to the activation of the MAPK pathway intracellularly, signaling of PI3K and src kinase and activation of STAT transcription factor (Yarden and Pines, 2012). The results indicate that the higher the expression of HSPA6, the more the number of genomic alterations, and the higher the degree of malignancy. However, glioma patients with IDH1 mutant tumors are known to have a better prognosis than those with the wild-type IDH gene (Yan et al., 2009). We also found that patients in the subgroup with higher HSPA6 levels possessed a higher TMB than those in the subgroup with low expression levels. Other researchers have already proven that some tumor patients can benefit from immunotherapy and that TMB can be used as a marker to predict the clinical response to immunotherapy (Cristescu et al., 2018).

Three of the six types of TIIC were found to be associated with HSPA6 CNV in patients with LGGs and GBMs. Previous studies showed that the degree of stromal and immune cell infiltration has a prominent effect on the prognosis of patients with tumors (Winslow et al., 2016). We generated an immune heat map that identified statistically significant correlations between HSPA6 expression and 29 immune-associated signatures and immune correlation scores, across the three cohorts. The higher the expression level of HSPA6, the higher the immune score, stromal score, and ESTIMATE score; the opposite effect was observed for tumor purity. Infiltrating immune cells are composed of microglia/macrophages, MDSCs, CD4^+^ T cells, regulatory T cells (Tregs), and granulocytes in the TME of gliomas. Infiltrating immune cells in the TME of gliomas consist of microglia/macrophages, CD4^+^ T cells, MDSCs, regulatory T cells (Tregs) and granulocytes; microglia and MDSCs are the most common factors (Gieryng et al., 2017); the presence of these factors is responsible for the inefficacious immune response in patients with glioblastoma (Marvel and Gabrilovich, 2015). HSPA6 was found to be involved in the development of cervical squamous cell carcinoma as an antigen processing and presentation gene (Qin et al., 2021). Zhang Peng et al. (Peng et al., 2021) also found HSPA6 as part of an immune signature in esophageal cancer (ESCA). Our results showed that various types of immune-associated cells had been produced in patients with glioma and that most of the immune checkpoints were significantly higher in the subgroup of patients with high expression levels of HSPA6.

Over recent years, immune checkpoint block (ICB) therapy has become a novel and effective form of treatment for various tumors (Pitt et al., 2016), particularly with regards to certain receptor molecules (CTLA-4 and PD-1). Our analyses showed that the levels of corresponding ligands (PDL1/PDL2 and CD80/CD86) were also significantly higher in the subgroup of patients with high expression levels of HSAP6. Therefore, it follows that a gaining a more enhanced understanding of the correlation between HSPA6 and immunity will inevitably yield new concepts for immunotherapy in patients with glioma.

## Conclusion

Herein, we described the biological functionality of HSPA6 in patients with glioma for the first time and verified our findings by performing *in vitro* experiments. However, our study had some limitations that need to be considered. Future research should investigate the precise immune and cellular functional mechanisms of HSPA6, both *in vivo* and *in vitro*. Whether HSPA6 can be regarded as an effective target-therapeutic agent for glioma needs to be investigated further.

## Data Availability

The datasets presented in this study can be found in online repositories. The names of the repository/repositories and accession number(s) can be found in the article/Supplementary Material.
