# Supplementary material for: HSPA6 is Correlated With the Malignant Progression and Immune Microenvironment of Gliomas
Source: Front Cell Dev Biol. 2022 Feb 23;10:833938. doi: 10.3389/fcell.2022.833938 (PMC8904718; doi:10.3389/fcell.2022.833938)
Supplement: Supplementary file 7 [file DataSheet1.docx]

**Figure legends**


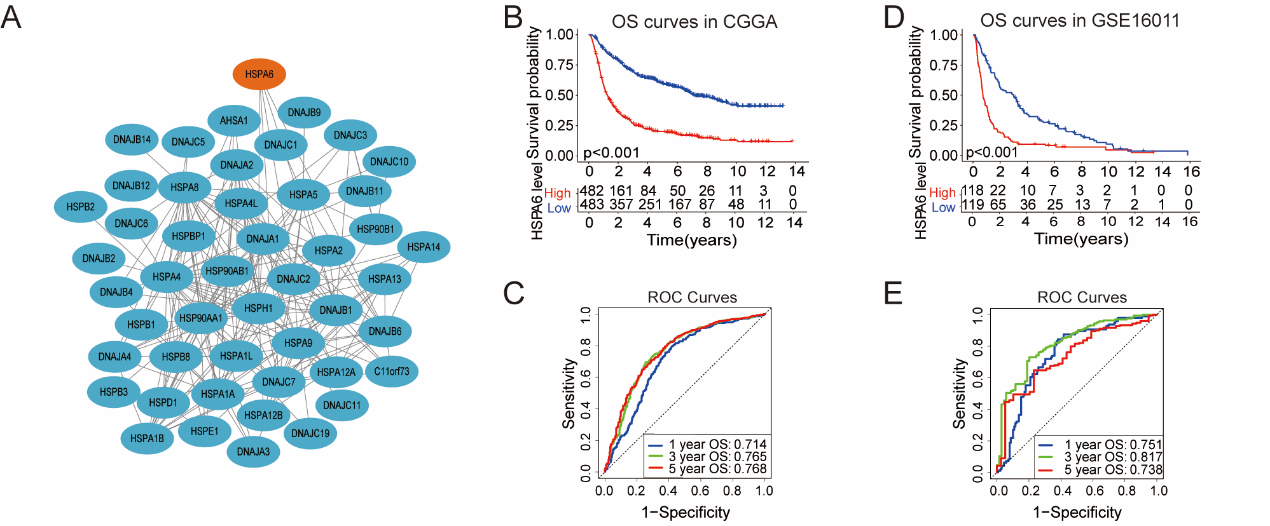


**FIGURE S1. PPI network, OS and ROC curves.** (A) A PPI network for members of the HSP superfamily was constructed by Cytoscape. (B-E) Kaplan–Meier and ROC-curve analyses revealed the significant prognostic value of HSPA6 based on the survival time of glioma patients in the CGGA and GSE16011 cohort.


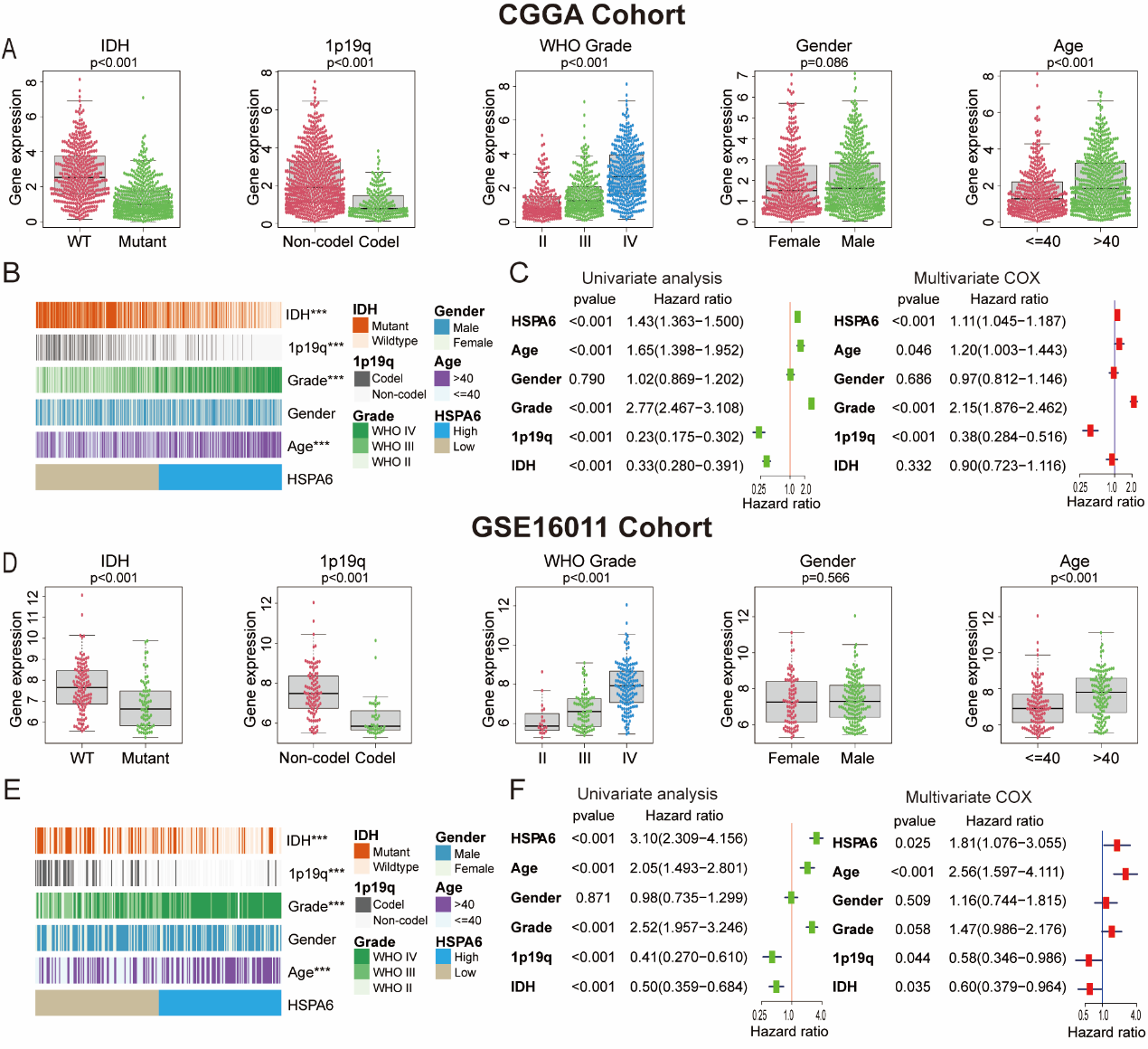


**FIGURE S2.** Relationship between HSPA6 expression profiles and clinical features in the CGGA (A, B) and GSE16011 (D, E) cohorts. ***P<0.001. Univariate and multivariate Cox regression analyses based on clinical features in the CGGA (C) and GSE16011 (F) cohorts.
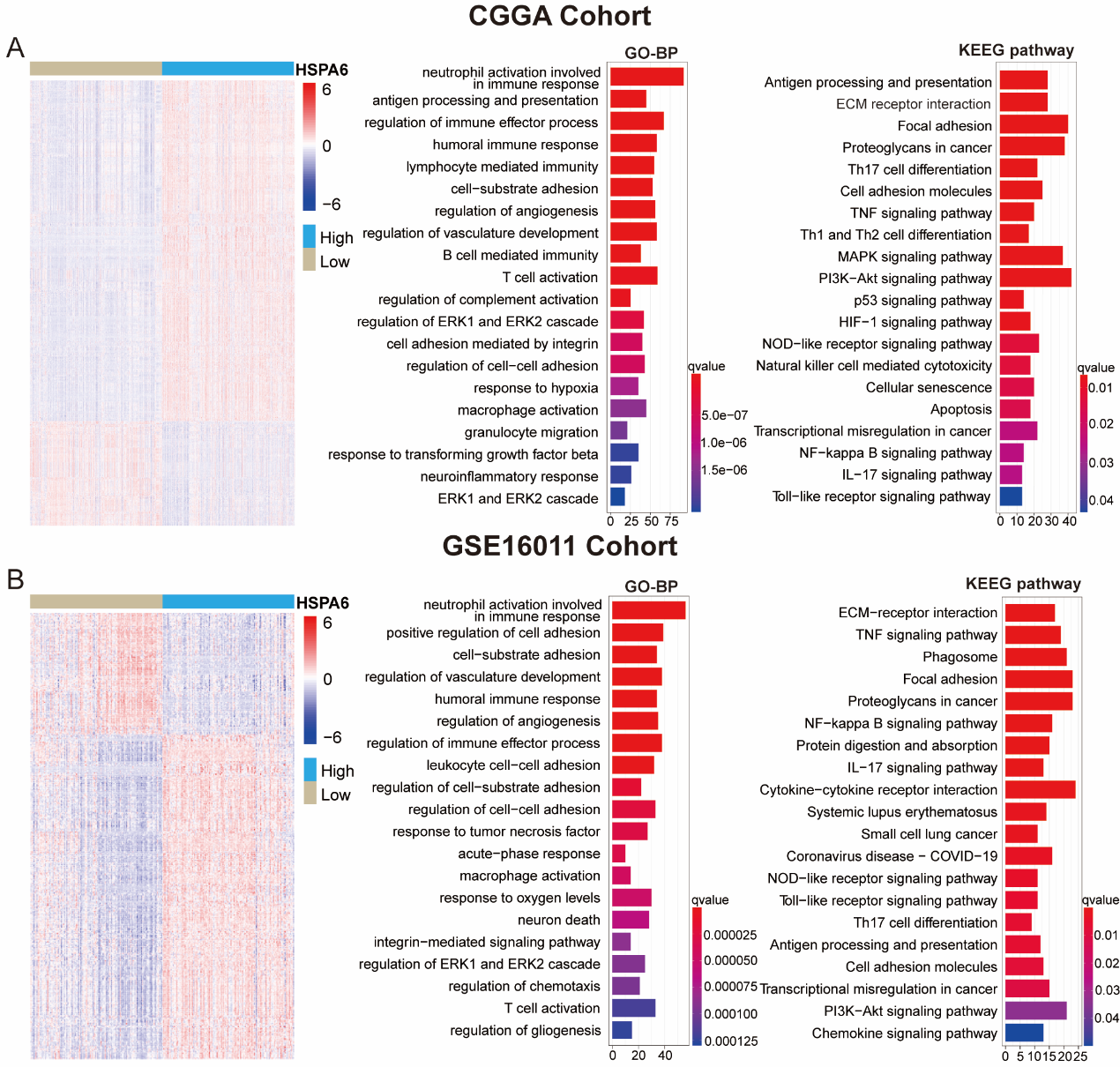


**FIGURE S3.** Functional annotation of high and low HSPA6 expression subgroups in the CGGA (A) and GSE16011 (B) cohorts.


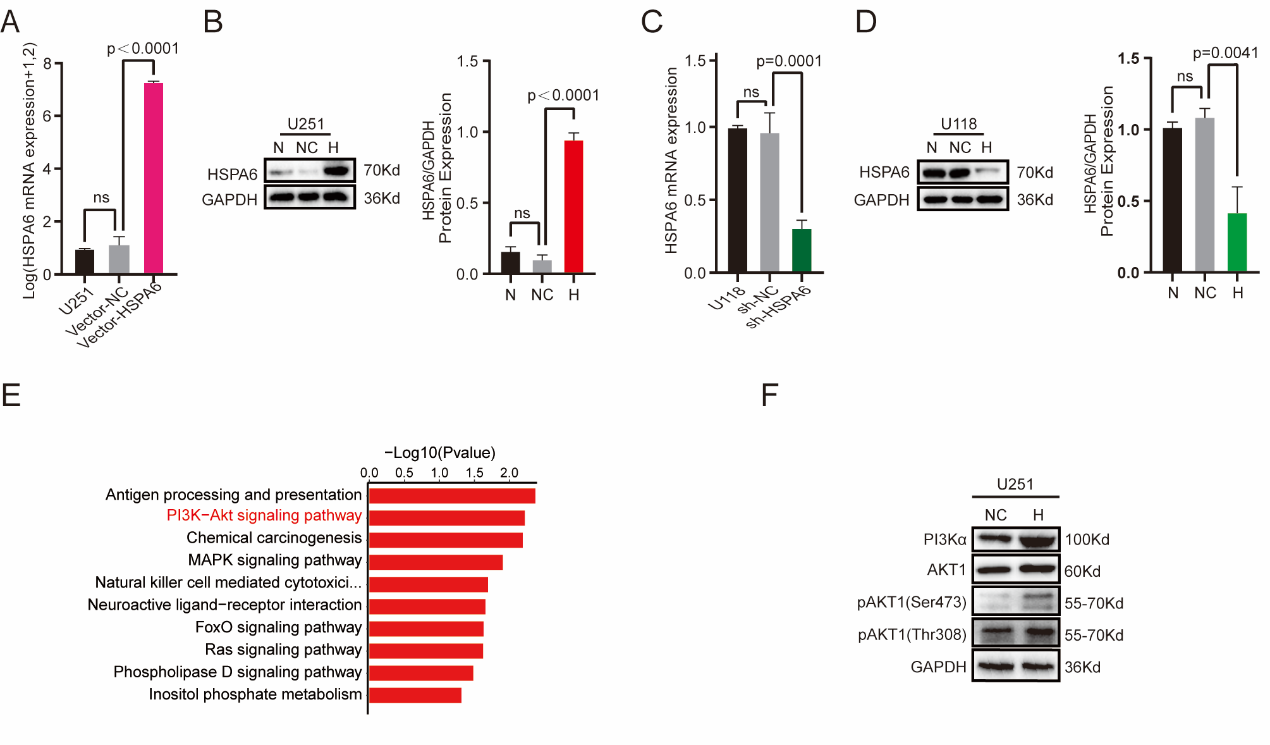


**FIGURE S4. The verification of HSPA6 overexpression or knockdown.** (A) Quantitative real time polymerase chain reaction (Q-PCR) detection of HSPA6 mRNA levels in U251 cells after transfection with a HSPA6 vectors compared with U251 normal cells and Vector-NC cells. (B) Western blotting detection of HSPA6 protein levels in U251 cells following transfection with the HSPA6 vector compared with U251 normal cells and Vector-NC cells. The expression levels of HSPA6 protein were quantified by ImageJ software. (C) qPCR detection of HSPA6 mRNA levels in 118 cells after transfection with sh-HSPA6 compared with U118 normal cells and sh-NC cells. (D) Western blotting detection of HSPA6 protein levels in U118 cells after transfection with sh-HSPA6 compared with U118 normal cells and sh-NC cells. HSPA6 protein expression levels were quantified by ImageJ software. Data are expressed as mean ± SEM from three independent experiments. ns, P > 0.05. (E). Pathway enrichment analysis of differential genes obtained by RNA sequencing. (F) Western blotting detection of PI3Kα, AKT1, and pAKT1 proteins levels in U251 cells following transfection with the HSPA6 vector compared with Vector-NC cells.


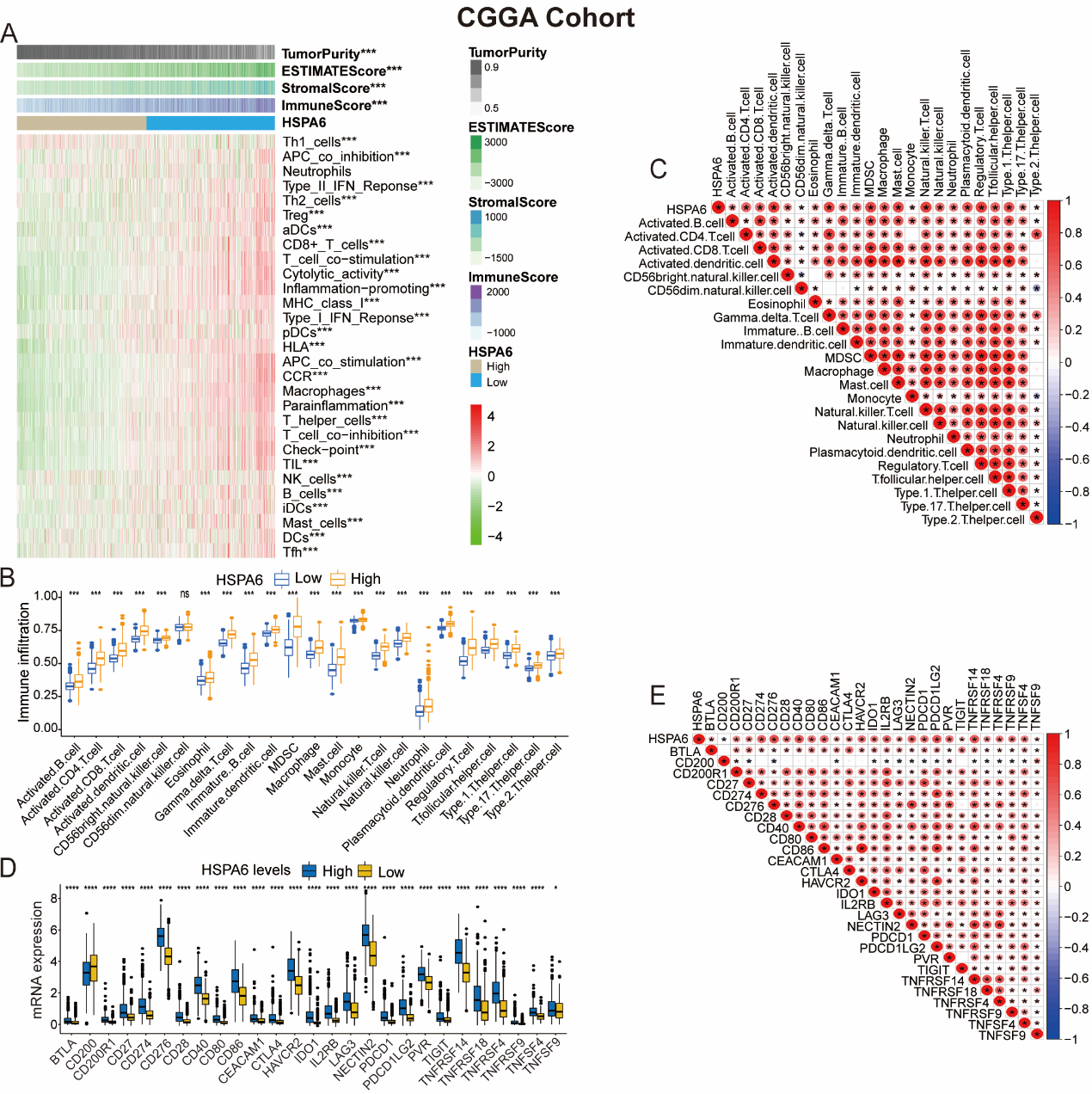


**FIGURE S5. Different TME characteristics in two subgroups from the CGGA cohort.** (A) Correlations of HSPA6 with 29 immune-associated gene sets, immune score, stromal score, ESTIMATE score, and tumor purity. (B) Different abundances of TIICs when compared between subgroups of patients with low and high expression levels of HSPA6. (C) Correlation analysis between HSPA6 and TIICs. (D) Differential analysis of the expression levels of ICPs between subgroups of patients showing low and high expression levels of HSPA6. (E) Correlation analysis between HSPA6 and the expression levels of twenty-seven immune checkpoints. ns, P > 0.05, * P < 0.05, ** P< 0.01, *** P< 0.001, ****P < 0.0001.


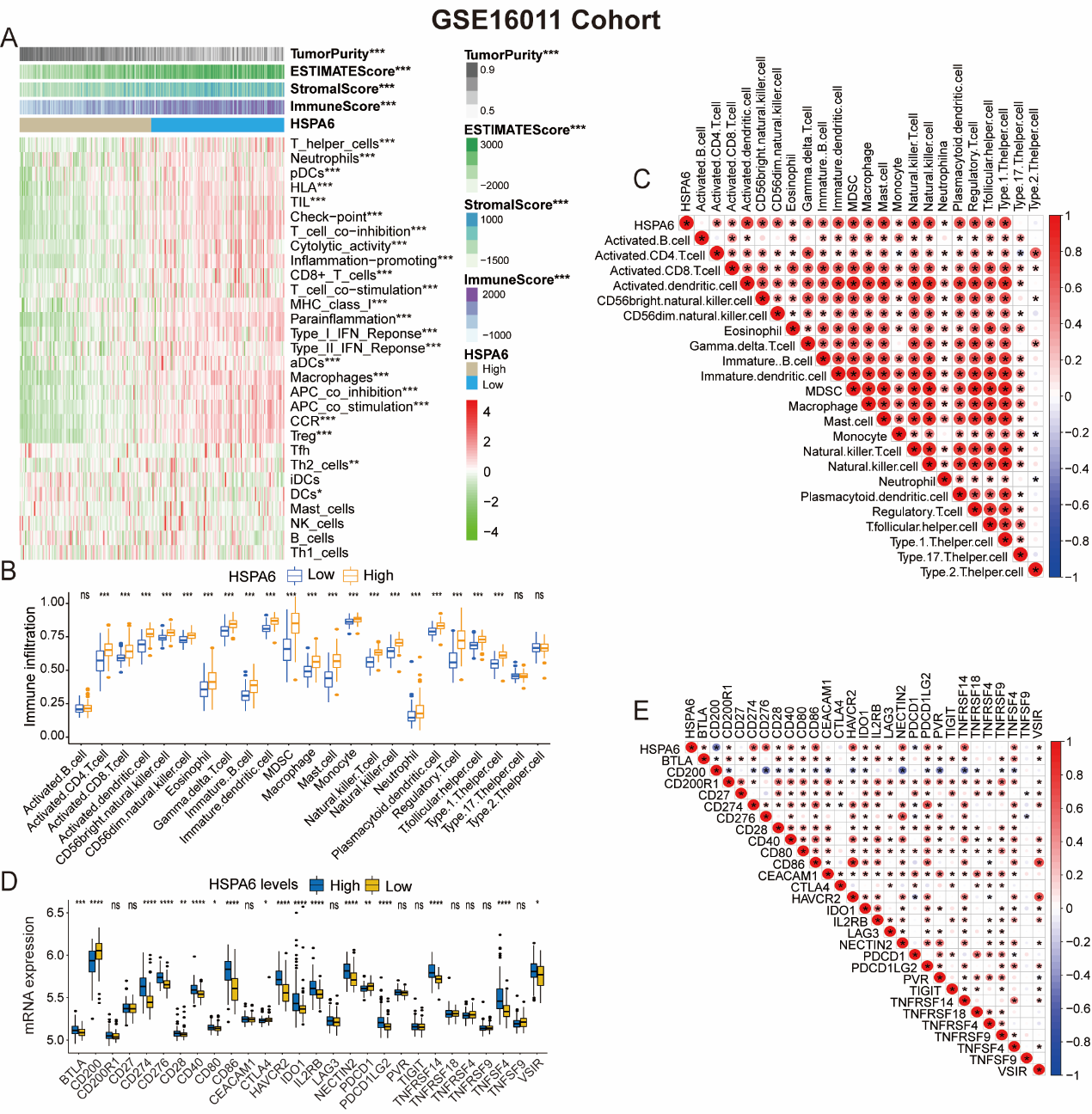


**FIGURE S6. Different TME characteristics in the two subgroups from the GSE16011 cohort.** (A) Correlations of HSPA6 with 29 immune-associated gene sets, immune score, stromal score, ESTIMATE score, and tumor purity. (B) Different abundances of TIICs between subgroups of patients with low and high expression levels of HSPA6. (C) Correlation analysis between HSPA6 and TIICs. (D) Differential analysis of several ICPs expression levels between subgroups of patients with low and high expression levels of HSPA6. (E) Correlation analysis between HSPA6 and the expression levels of twenty-seven immune checkpoints. ns, P > 0.05, *P < 0.05, **P< 0.01, ***P< 0.001, ****P < 0.0001.
